# Quality parameters for RNA preparations from biopsies of ulcerated human skin

**DOI:** 10.12688/wellcomeopenres.18052.1

**Published:** 2022-10-05

**Authors:** Lina Giraldo-Parra, Lady Giovanna Ramirez, Adriana Navas, María Adelaida Gómez

**Affiliations:** 1 Centro Internacional de Entrenamiento e Investigaciones Médicas-CIDEIM, Cali, Valle del Cauca, 760031, Colombia; 2 Universidad Icesi, Cali, Valle del Cauca, 760031, Colombia; 3 Department of Internal Medicine and Radboud Center for Infectious Diseases, Radboud University Nijmegen Medical Centre, Nijmegen, The Netherlands

**Keywords:** skin biopsies, Allprotect®, RNA, gene expression, cutaneous leishmaniasis, skin ulcer

## Abstract

**Background: **Obtaining high quality RNA from skin biopsies is complex due the physical composition and high content of nucleases of this tissue. This becomes particularly challenging when using compromised skin samples with necrotic, inflammed or damaged areas, such as those from patients suffering skin conditions, which affect more than 900 million people annually. We evaluated the impact of the biopsy size and tissue preservation method on the quality and quantity of RNA extracts.

**Methods:** Skin lesion biopsies were obtained from patients with cutaneous leishmaniasis (CL). Biopsy specimens of 2 mm (n = 10) and 3 mm (n = 59) were preserved in Allprotect® reagent, and 4 mm biopsies in OCT (n = 54). Quality parameters were evaluated using Nanodrop and Bioanalyzer. The informativeness of the extracted samples for downstream analyses was evaluated using RT-qPCR and RNA-Seq.

**Results: **The success rate, based on quality parameters of RNA extraction from tissue biopsies stored in OCT and 2 mm biopsies stored in Allprotect®, was 56% (30/54) and 30% (3/10), respectively. For 3 mm skin biopsies stored in Allprotect® was 93% (55/59). RNA preparations from 3 mm-Allprotect® biopsies had an average RIN of 7.2 ± 0.7, and their integrity was not impacted by sample storage time (up to 200 days at -20°C). RNA products were appropriate for qRT-PCR and RNA-seq. Based on these results, we propose a standardized method for RNA extraction from disrupted skin samples. This protocol was validated with lesion biopsies from CL patients (n = 30), having a success rate of 100%.

**Conclusions: **Our results indicate that a biopsy size of 3 mm in diameter and preservation in Allprotect® for up to 200 days at -20°C, are best to obtain high quality RNA preparations from ulcerated skin lesion biopsy samples.

## Introduction

The skin is the principal anatomical barrier for protection against environmental exposure to microorganisms, xenobiotics, and other insults that could damage the host ^1^. Although the skin is one of the most accessible clinical samples, it is a defiant tissue for RNA/DNA-based research because it contains a high level of nucleases, rendering nucleic acids highly susceptible to degradation. Due to the stability of the hyaluronic acid-collagen matrix ^2^, skin disruption and homogenization require mechanical and chemical forces that can damage nucleic acids. Therefore, obtaining high-quality nucleic acids becomes particularly challenging from tissues such as ulcerated skin, and thus downstream analyses are often minimally informative or biased by the method used for DNA and RNA preparations.

Tissue samples with necrotic or damaged areas due to infectious diseases or trauma, or with substantial inflammatory infiltrate and/or edema, are prone to high DNA/RNA degradation. This is the case of dermal lesion samples from patients with skin conditions caused by chronic non-communicable or infectious diseases, which affect over a billion people globally each year ^3^. Among these are a number of neglected tropical skin diseases, of which cutaneous leishmaniasis (CL) contributes to more than one million cases annually ^4, 5^. We aimed to provide a standardized protocol for obtaining good quality RNA from inflamed skin tissue, and to describe the most appropriate conditions for sample collection, storage, and RNA extraction from human samples.

## Methods

***Ethics approval:*** Study protocols, consent forms, and all procedures were approved by Centro Internacional de Entrenamiento e Investigaciones Médicas (CIDEIM) Institutional Review Board for the ethical conduct of research involving human subjects, in compliance with national (resolution 008430, República de Colombia, Ministry of Health, 1993) and international (Declaration of Helsinki and amendments, World Medical Association, Fortaleza, Brazil, October 2013) guidelines. All participants provided written informed consent.

**Figure 1.**
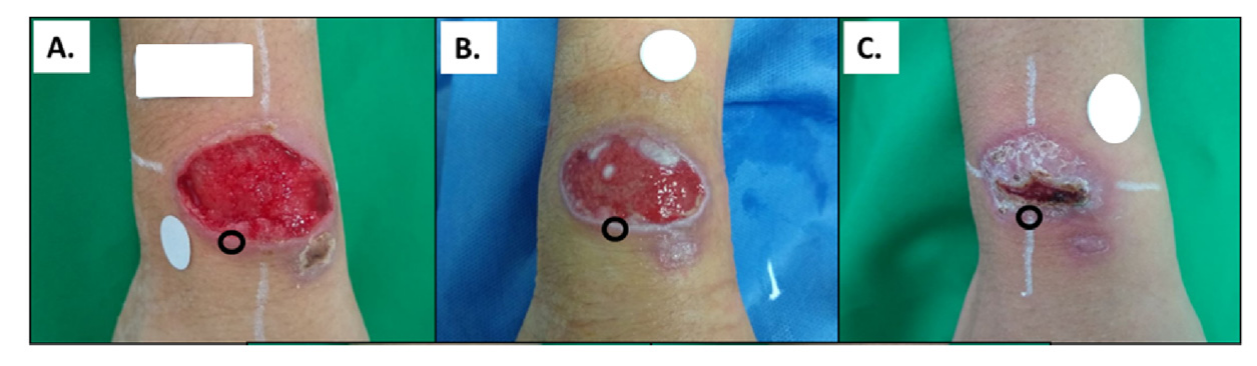
Representative images of the evolution of a CL lesion in a patient during the course of treatment with meglumine antimoniate. **A**. Typical CL ulcer (pre-treatment). **B**. Lesion midway through treatment (day 10). **C**. Lesion at end-of-treatment (day 20). Black circles indicate the site where skin biopsies were obtained. These pictures were obtained from the same patient. Source: Clinical Unit, CIDEIM.

***Samples*:** This study was based on a secondary analysis of data from skin biopsy samples from patients with parasitological confirmation of CL recruited as part of two projects carried out during different periods at CIDEIM in the cities of Cali and Tumaco, Colombia. The samples were divided into two groups according to the reagent used for tissue storage. The****** first group (Group 1) included 54 lesion biopsies from 27 CL patients enrolled between the years 2009–2015. Lesion biopsies were obtained from patients before initiation of antileishmanial treatment with meglumine antimoniate (n = 27) and at the end of treatment (n = 27). Group 2 included 69 biopsies of CL lesions from 24 patients enrolled between the years 2016–2019. Sequential biopsies from the same lesion of each patient were obtained before (n = 24), in the middle (n = 24), and at the end of treatment (n = 21). Group 3 was defined as a validation group, which included 30 lesion biopsies obtained before treatment from 30 CL patients. Skin biopsies were taken under local anesthetic, and one sample was taken at a time. The punch biopsy was obtained considering the following ratio: 1/3 of healthy skin and 2/3 of the edge of the lesion (the indurated edge did not include necrotic tissue) ( Figure 1A–C).

**Figure 2.**
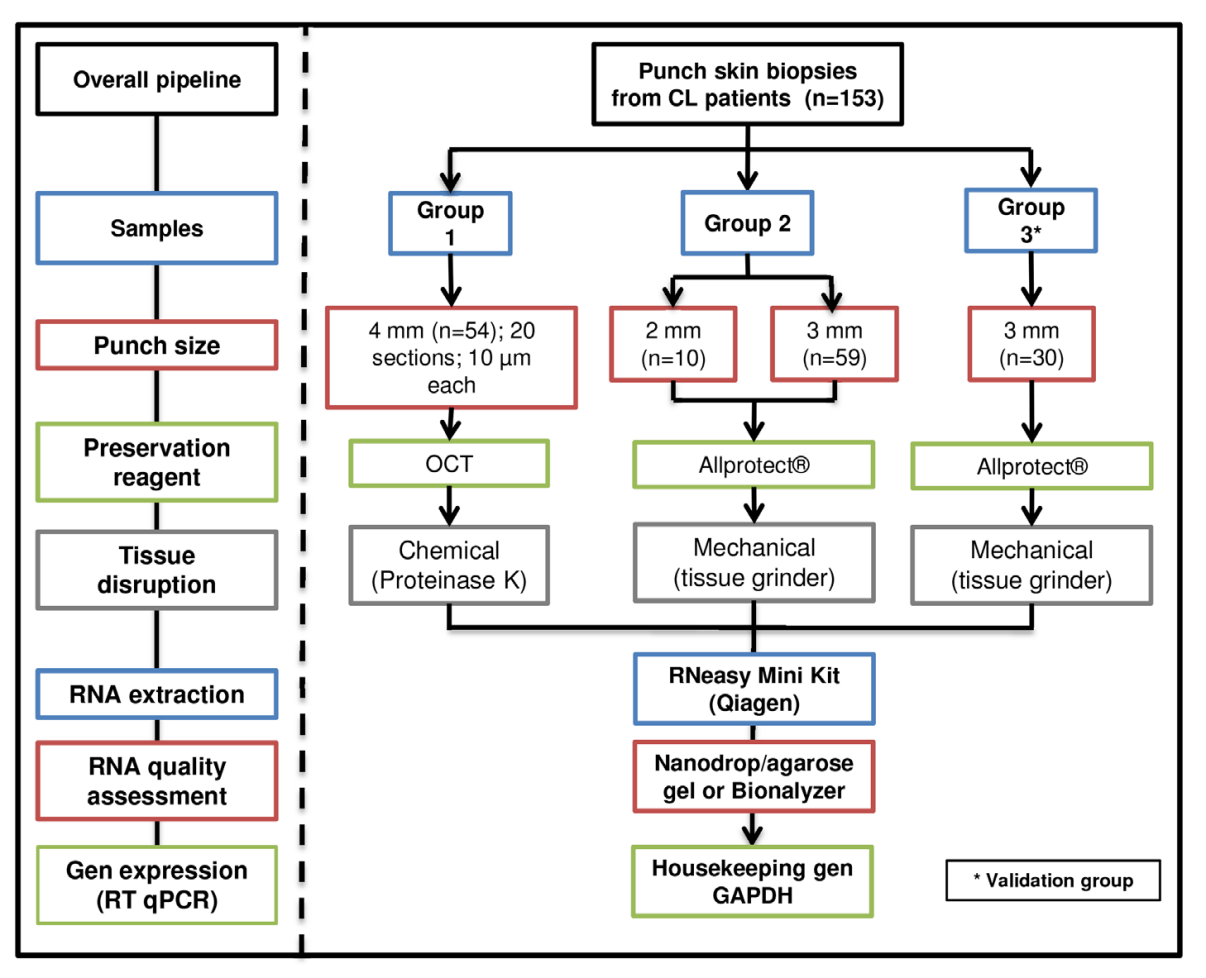
Schematic workflow. Skin lesion biopsies were obtained from two cohorts of CL patients. The size of the biopsy, preservation and disruption methods are shown.

***Tissue processing***: Overall summary of skin biopsy collection, preservation, disruption, homogenization, and RNA extraction for the groups ( Figure 2).

Group 1: Punch biopsies of 4 mm were frozen in Tissue-Tek™ OCT Compound (Sakura Finetek Europe B.V # 4583) and cryopreserved in liquid nitrogen until processing. For evaluation of gene expression, 20 tissue sections of 10 µm each were obtained before RNA extraction and stored back in liquid nitrogen. The sections were removed from the nitrogen tank and immediately homogenized by strong vortexing for 1 min with RLT buffer (RNeasy Mini Kit, Qiagen cat. 74004, Valencia, CA). Due to the high protein content of the skin, the sections were subjected to enzymatic denaturation with proteinase K (Qiagen cat. 19133, Valencia CA) for 10 minutes at 55°C. RNA was extracted using the RNeasy Mini Kit, following the manufacturer's protocol and using DNAse digestion (Qiagen, cat. 79254) for the removal of contaminating genomic DNA. RNA was eluted from the RNAeasy column in 14 µL of RNase-free water (Qiagen) and stored at -80°C until further use. For cDNA synthesis, RNA (100 ng) was transcribed with the High-capacity cDNA reverse transcription kit (Applied Biosystems, Foster City, CA). Gene expression was measured in real-time with the CFX96 Real-Time System (RRID:SCR_018064) (Bio-Rad, Hercules, CA) using Taqman Universal PCR master mix (Applied biosystem, cat. 4304437) and Taqman gene expression assays for *GAPDH* gene (Hs99999905_m1) (Thermofisher Scientific), with the following protocol: denaturation at 95°C for 10 minutes, followed by 49 cycles of 95°C for 15 seconds, and 60°C for 1 minute, or using PCR arrays (Qiagen, ref No. PAHS-077Z) that included the GAPDH gene (PPH00150F) and RT² SYBR green qPCR mastermix (Qiagen, cat. 1065806), with the following protocol: denaturation at 95°C for 10 minutes, followed by 40 cycles of 95°C for 15 seconds, and 60°C for 1 minute. Ct values were analyzed in the CFX Manager ^TM^ 3.1 software #1845000 (Bio-Rad) (RRID:SCR_017251).

Group 2: Skin biopsies of 2 mm (n = 10) and 3 mm (n = 59) were taken and immediately stored in 1 mL Allprotect® (Qiagen). Samples were equilibrated overnight at 4°C and then stored at -20°C until processing. The tissue sample was cut into smaller pieces with sterile scalpel blades, followed by tissue disruption and homogenization using a manual glass tissue grinder on an ice bath containing Trizol® for no more than 5 minutes ( https://dx.doi.org/10.17504/protocols.io.x54v9yn7zg3e/v1). The lysate was centrifuged together with chloroform solution at 13000 rpm for 15 minutes, and the aqueous phase was mixed with ethanol and passed through RNA purification columns (RNeasy Mini Kit, Qiagen), following the manufacturer's instructions. RNA was eluted from the RNAeasy column in 25 µL of RNase-free water (Qiagen) and stored at -80°C until it was used for analysis. cDNA was synthesized using the RT first-strand synthesis kit (Qiagen, cat. 330404). RT-qPCR reactions were run on a CFX96 Real-Time System (RRID:SCR_018064) (Bio-Rad) using Taqman Universal PCR master mix (Applied biosystem, cat. 4304437) and Taqman gene expression assays for *GAPDH* gene (Hs99999905_m1) (Thermofisher Scientific), with the following protocol: denaturation at 95°C for 10 minutes, followed by 49 cycles of 95°C for 15 seconds, and 60°C for 1 minute or using custom made PCR arrays (Qiagen, ref No. CLAH23658D) that included the GAPDH gene (PPH00150F) and RT² SYBR green qPCR mastermix (Qiagen, cat. 1065806), with the following protocol: denaturation at 95°C for 10 minutes, followed by 40 cycles of 95°C for 15 seconds, and 60°C for 1 minute. Ct values were analyzed in the CFX Manager ^TM^ 3.1 software #1845000 (Bio-Rad) (RRID:SCR_017251).

Group 3 (validation group): Skin biopsies of 3 mm (n = 30) were taken and immediately stored in 1 mL Allprotect® (Qiagen). Samples were processed with the RNA extraction protocol described for group 2, and five samples were used for RNAseq.

***RNA quality assessment*:** The quantity and purity of the extracted RNA was evaluated in a Nanodrop ND-1000 spectrophotometer (RRID:SCR_016517). The acceptable ranges of absorbance ratios at 260/280 and 260/230 were 1.8–2.0 and 1.8–2.2, respectively. For the samples stored in OCT, the RNA integrity was evaluated by the presence of two intact ribosomal bands (18S and 28S) in agarose gel electrophoresis (1.3%). For samples stored in Allprotect®, this was assessed using an Agilent 2100 Bioanalyzer (RRID:SCR_018043) (RNA 6000 Nano LabChip, Agilent Technologies); an RNA integrity number (RIN) value of ≥7 was defined as good quality RNA.

***RNA-Seq*:** Construction of cDNA libraries was performed using the Illumina TruSeq® Stranded mRNA kit V2. The quality evaluation and quantification of the libraries were carried out using the Agilent 2100 Bioanalyzer (RRID:SCR_018043) (DNA 1000 kit or High Sensitivity DNA Assay, Agilent Technologies). The samples were sent to the University of Maryland Bioscience and Biotechnology Research Institute for pair-end 100bp Illumina HiSeq RNA-seq. The quality control of the sequences and the assembly were performed using standard pipelines developed for CL lesion transcriptomic analyses ^6^. Bioinformatic deconvolution of transcriptomes (n=5) was performed using xCell ^7^.

***Statistical analyses*:** The Kolmogorov-Smirnov and Shapiro-Wilk tests were used to determine the parametric or nonparametric distribution of the data. One-way analysis of variance (ANOVA) or Kruskal-Wallis test followed by multiple-comparison tests were employed for group comparisons. For nonparametric data, comparisons were analyzed using the Wilcoxon signed-rank test. Statistical significance was defined at a *p*-value < 0.05. All data were analyzed using GraphPad Prism 6 software (RRID:SCR_002798), (statistical analyses can be performed in open-access software such as the R Stats package *v.4.2.1*).

## Results and discussion

RNA quality is essential for obtaining meaningful and reliable results from gene expression data, even more when samples are irreplaceable such as those collected from human participants of clinical studies. Here, we report the quality characteristics of RNA samples obtained from skin biopsies from ulcerated cutaneous lesions from CL patients, which were preserved and processed using different commercially available protocols ^8^. Based on a comparative analysis, we then propose a standardized method for preservation, processing, and RNA extraction of ulcerated skin lesion samples.

***The size of the skin biopsy rather than the sample preservation method determines the quantity of RNA extracted from punch biopsy samples.*** Obtaining high-quality RNA in sufficient quantity from small skin biopsy samples is challenging due to the alteration of the tissue and the activation of endogenous RNases, which makes the skin one of the most difficult tissues from which to obtain intact RNA. Initially, large amounts of frozen skin tissue were used (>100 mg) ^9^ and downsized to less than 10 mg of tissue per sample ^10^. Previous studies in mice have shown that the minimum size of a skin biopsy to consistently obtain sufficient RNA for transcriptomics experimentation was a 1.5 mm skin punch biopsy ^10^. However, these results were based on intact skin samples. In our study, we address one additional difficulty: how to obtain good quality and good quantity RNA samples for downstream gene expression analyses from compromised human skin tissue such as those from CL patients whose lesions are highly inflamed and ulcerated.

**Figure 3.**
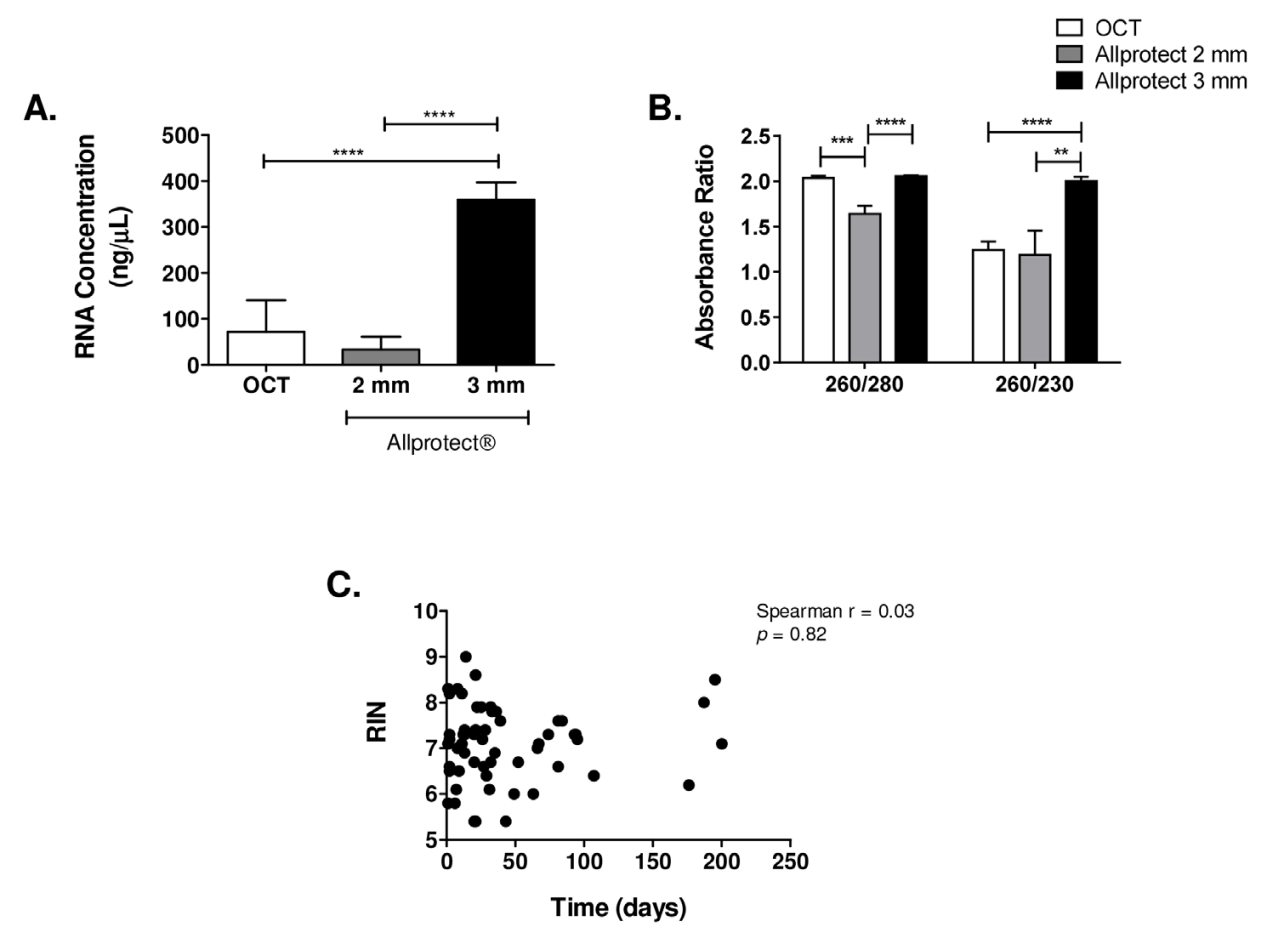
Effect of skin biopsy size and preservation solution on RNA quality and quantity. **A**. RNA concentration (ng/µL). **B**. Absorbance ratios 260/280 and 260/230. Data are presented as the mean ± SEM and statistical significance was estimated by Kruskal-Wallis test. **: *p* < 0.01; ***: *p* < 0.001, ****: *p* < 0.0001. OCT- 4 mm (n=54), Allprotect®-2 mm (n=10) and Allprotect®-3 mm (n=59). **C**. Effect of sample storage time on the RNA integrity based on RIN. The Spearman’s correlation was used to evaluate the relationship.

RNA concentrations obtained from processing the entire 2 mm biopsies stored in Allprotect®, and the 20 sections of 10 µm each of the 4 mm biopsies stored in OCT (equivalent to processing 2 mm of the biopsy), were comparable ( Figure 3A) and together were significantly lower than those from total tissue extracts of 3 mm biopsies stored in Allprotect® ( *p* <0.0001). This shows that the amount of tissue, rather than the preservation medium, is the principal factor affecting the RNA quantity in skin biopsy extracts and that lesion skin biopsies with a size < 3 mm should not be used for samples which include areas of high inflammatory infiltrate, edema or necrosis.

***RNA samples from 3 mm skin biopsies stored in Allprotect* **®** *outperform in terms of sample purity and RNA integrity.*** RNA samples without protein or solvent contamination should have a 260/280 absorbance ratio of 1.8–2.0 and 260/230 of 2.0–2.2, respectively. Tissues preserved in OCT and Allprotect® (3 mm) had good and comparable 260/280 ratios: 2.04 and 2.06, respectively. Biopsies of 2 mm stored in Allprotect® had a significantly lower 260/280 ratio of 1.64 ( Figure 3B). Only 3 mm-Allprotect® samples had a 260/230 absorbance ratio ~2.0 ( Figure 3B). According to these results, the 3 mm-Allprotect® samples were the only RNA extracts complying with the two indices of nucleic acid purity.

The integrity of RNA from lesion biopsies stored in Allprotect® was analyzed using the Agilent Bioanalyzer and agarose gels. The presence of intact ribosomal RNA bands was observed in 3/10 biopsies from the 2 mm-Allprotect® group with an average RIN of 6.2 ± 0.2, and in 55 of the 59 3mm-Allprotect® biopsies samples, with an average RIN of 7.2 ± 0.7. To control the possible effect of tissue storage time at -20°C on RNA integrity, we analyzed the correlation between a time range of 1 to 200 days of storage in Allprotect® at -20°C and the RIN values. No significant correlation was found (Spearman r = 0.03, *p* = 0.82) ( Figure 3C), indicating that RNA integrity was not impacted by sample storage time.

Several commercial RNA stabilization reagents are available for tissue preservation, minimizing the requirement of immediate sample processing and freezing. These systems allow the collection and shipment of samples from rural areas to central laboratories without the requirement of complex shipping conditions, such as liquid nitrogen tanks. Skin tissue preservation in Allprotect® offers the advantage of avoiding intermediate tissue freezing compared to OCT. Other studies have also demonstrated effective stabilization of tissue from primary colorectal tumor samples, breast cancer, and colorectal liver metastases with Allprotect®, resulting in high-quality isolated DNA, RNA, and proteins when compared to samples preserved in liquid nitrogen ^11, 12^. However, some RNA degradation has been documented in tissues preserved for one week in Allprotect® at 8°C, and protein degradation after two weeks ^11^. In a comparative study using pig skin, biopsies were immediately stored as freeze-dry biopsies, or stored in Allprotect®, QIAzol lysis reagent, or RLT lysis buffer containing beta-mercaptoethanol ^13^. All four strategies resulted in RNA with high RIN values (≥ 8.5); however, samples in Allprotect® and QIAzol had lower OD 260/230 indices ^13^, which we also observed for human ulcerated skin samples preserved in Allprotect®.

***Effects of RNA preparations on downstream applications.*** The success rate of RNA extraction was defined according to the number of samples that met the quality parameters (integrity and purity thresholds) over the total number of samples corresponding to their study group ( Table 1). The success rate of RNA extraction from tissue biopsies stored in OCT and 2 mm biopsies stored in Allprotect® was 56% (30/54) and 30% (3/10), respectively. The success rate of 3 mm skin biopsies stored in Allprotect® was 93% (55/59). RT-qPCR was used to assess whether RNA extracts obtained by each of the three biopsy sample types were sufficient for downstream applications. Average Ct values for GAPDH (amplification product average size of 122 bp for Taqman and 130 bp for PCR arrays) from samples deemed successful ( Table 1) were similar in samples from all groups (OCT-4 mm: 24.6 ± 1.1 (n = 27); Allprotect®-2 mm: 24.3 ± 2.0 (n = 3); and Allprotect®-3 mm: 25.7 ± 1.3 (n = 50)). This indicates that despite suboptimal RNA preparations obtained from 2mm-Allprotect® biopsies and 4mm-OCT biopsies, amplification of short RNA sequences is feasible and comparable.

**Table 1.** Quality scores based on RNA purity and integrity parameters. The quantity and quality of RNA isolated utilizing different preservation reagents. Data are presented as mean ± SD.

Quality metrics of successful samples							
Diameter (mm)	Preservation reagent	Success rate (%)	Nanodrop (ng/µL)	Bioanalyzer (ng/µL)	A260/280	A260/230	RIN
2	Allprotect®	30 (3/10)	59.9 ± 36.9	70.3 ± 44.7	1.91 ± 0.06	1.67 ± 0.60	6.2± 0.2
3	Allprotect®	93 (55/59)	384.0 ± 280.2	365.1 ± 251.9	2.06 ± 0.04	2.08 ± 0.20	7.2 ± 0.7
4	OCT *	56 (30/54)	100.7 ± 73.8	----	2.05 ± 0.09	1.75 ± 0.27	----

* Samples preserved in OCT have no assessment of RNA quality using Agilent Bioanalyzer 2100.

***Suggested protocol and validation.*** Based on the above results, we include a suggested standard protocol for working with ulcerated skin samples for RNA processing ( https://dx.doi.org/10.17504/protocols.io.x54v9yn7zg3e/v1). A new cohort of 30 CL patients from which 3 mm lesion biopsy samples were obtained, was evaluated as a validation cohort for the suggested protocol, achieving a success rate of 100% (30/30), based on the integrity parameters defined above. The average concentration of RNA was 527.5 ± 368.2 ng/µL, the 260/280 and 260/230 indexes were 2.05 ± 0.04 and 2.18 ± 0.09, respectively, and RIN 7.1 ± 1.

**Figure 4.**
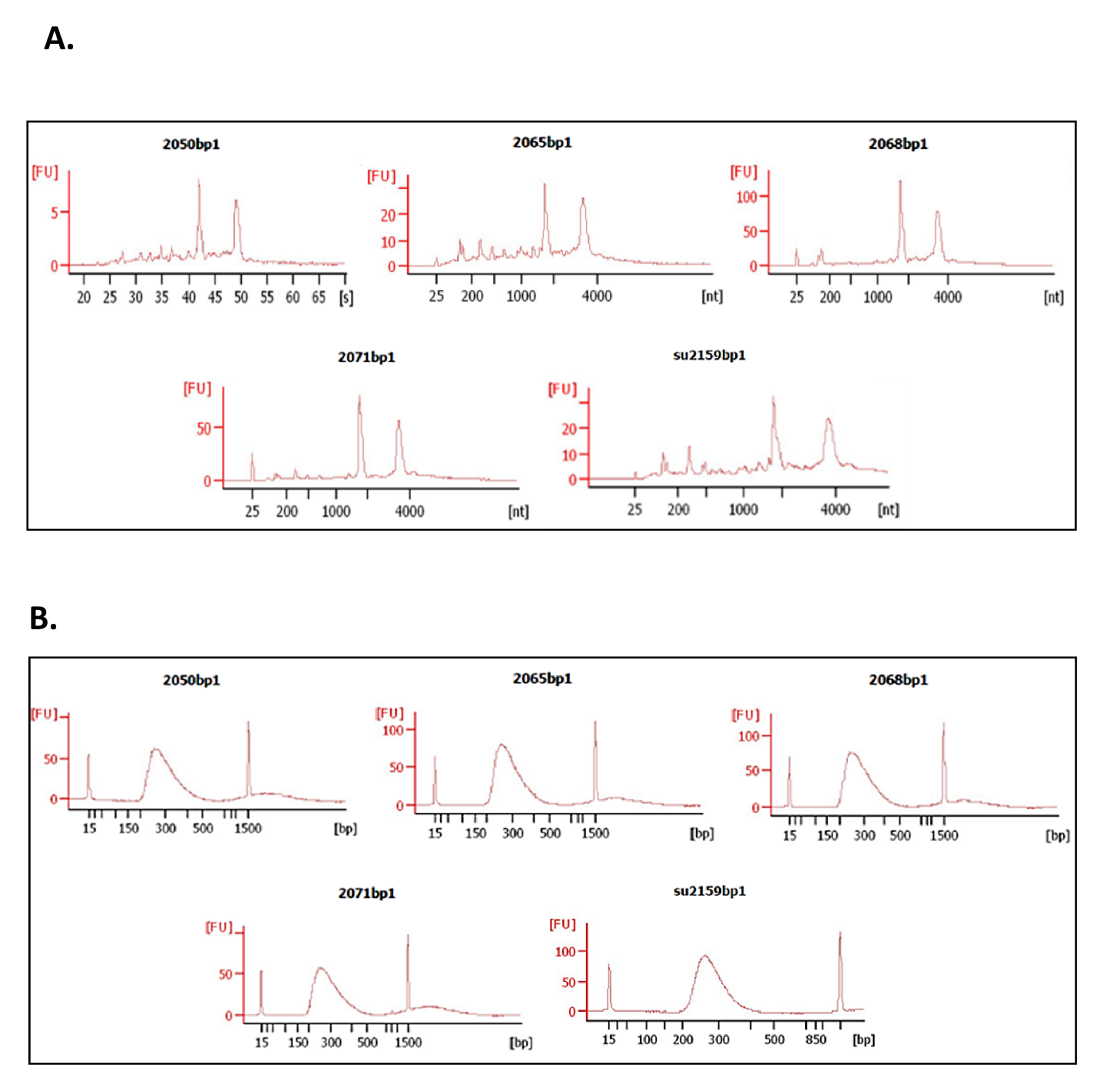
Quality assessment of RNA and cDNA libraries using Bioanalyzer Agilent 2100. **A**. Electropherogram shows two peaks corresponding to the ribosomal subunits 18s and 28s (from left to right). **B**. Electropherogram of the 5 library samples constructed with the Illumina TruSeq® Stranded mRNA kit. Average library size approximately ~ 286 bp.

As a proof-of-principle of the adequacy of 3 mm skin biopsy samples preserved in Allprotect® for in-depth RNA-based processes, cDNA libraries were constructed from RNA samples from lesions of 5 CL patients infected with *L (V). panamensis* ( Figure 4A). cDNA libraries showed an average size of 286 ± 5 bp with a concentration of 157.6 ± 18.6 nM ( Figure 4B). The number of reads obtained from these samples ranged from 5.771.497 to 12.921.744. The reads aligned to both human (hg38) and *L.V. panamensis* (v36) genomes using HISAT2.2.1 (RRID:SCR_015530). On average, 93.8 ± 2.1% of the total number of reads aligned to the human genome, while 1.2 ± 1.5% to *L. V. panamensis*.

**Figure 5.**
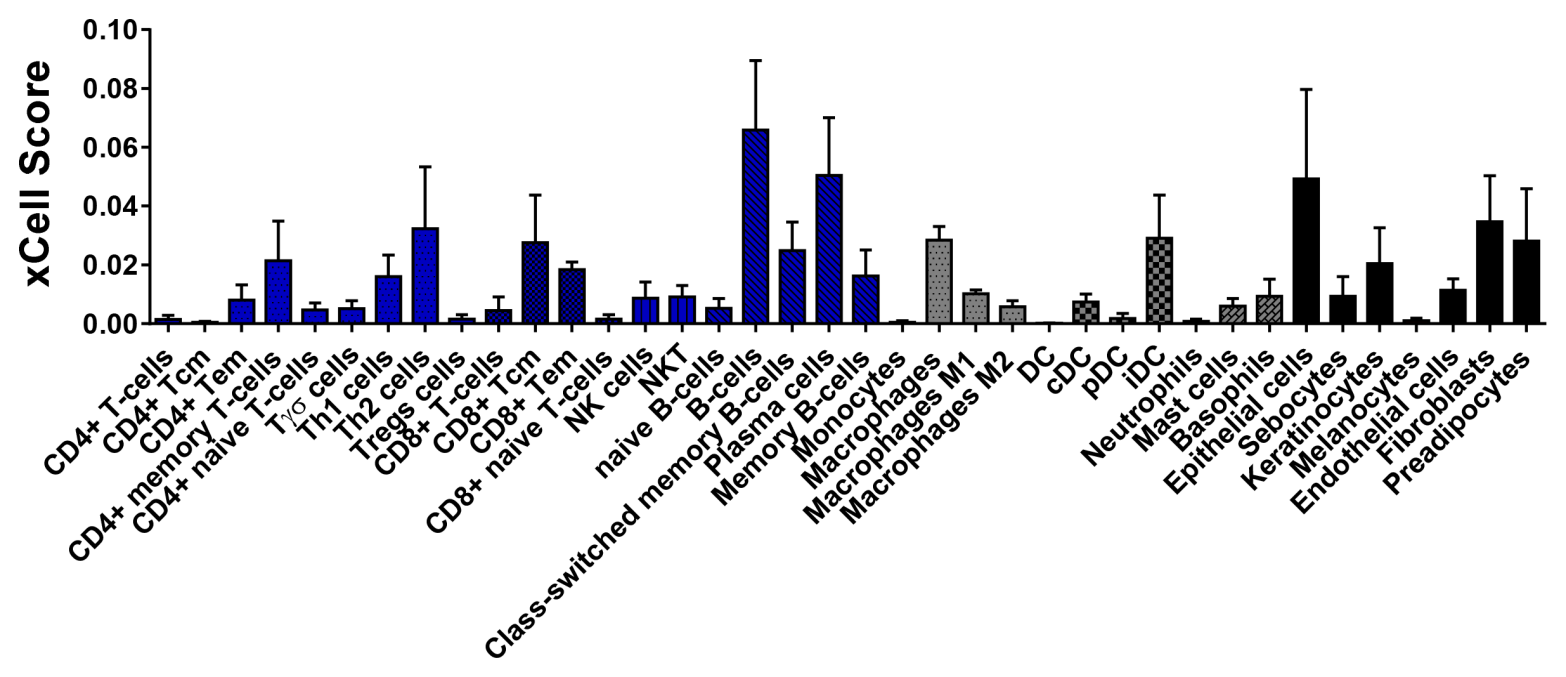
Estimate of immune response cells in lesion biopsies from CL patients before treatment. xCell was used to estimate the cell subsets in pre-treatment biopsies from CL patiens based on RNA-Seq data (n = 5). The biopsies were preserved in Allprotect® reagent. The data are represented as xCell scores.

We performed a deconvolution analysis to infer the immune cell composition of the tissues using xCell ^7^, and compared this to previously published immune cell characterization of CL lesions ^14, 15^. Past characterization studies of the cell populations present in lesions of CL patients ^16– 18^, have been based on immunohistochemistry and flow cytometry, which are restricted to the study of a small group of cells. Through deconvolution analysis of RNA-Seq data, we pursued an alternative method to overcome this limitation. xCell performs cell-type enrichment analysis from gene expression data for 64 immune cells and stromal types, generating a score that refers to the estimate of the cell population ^7^. Bioinformatic results show that CL lesions were characterized by the presence of structural skin cells (epithelial cells, keratinocytes, fibroblasts), and an important infiltrate of immune cells which included B cells (memory and plasma cells), lymphocyte subpopulations CD4+ (memory, Th1, Th2, Tγδ, and Tregs), CD8+ (central memory and effector cells), followed by macrophages and dendritic cells ( Figure 5). A immunohistochemistry study by Guarin *et al*. ^15^ showed that acute lesions from CL patients presented a cellular infiltrate with a predominance of mononuclear cells that include macrophages, CD4+ and CD8+ T lymphocytes, and B lymphocytes, and a small proportion of polymorphonuclear cells including eosinophils ^15^. Our characterization of cell populations by deconvolution analysis shows the participation of the same cell populations, however, a broader picture can be obtained including subpopulations for some of these cell lineages (eg. B cells (memory and plasma cells), lymphocyte subpopulations CD4+ (memory, Th1, Th2, Tγδ, and Tregs), and CD8+ (central memory and effector cells). These subpopulations have also been reported in targetted phenotypic analyses of CL lesions ^19, 20^.

## Conclusions

In conclusion, we present a validated methodology to obtain good quality RNA from ulcerated skin samples for use in translational research studies.

## Consent

Written informed consent for publication of the patients’ details and their images was obtained from the patients.

## Data Availability

Open Science Framework: Underlying data for ‘Quality parameters for RNA preparations from biopsies of ulcerated human skin’ https://doi.org/10.17605/OSF.IO/CKF3G ^8^ This project contains the following underlying data: Data file 1: Raw data.xlsx (RNA concentrations, absorbance, RIN values and Ct values (GAPDH)). Data file 2: Bioanalyzer files. Data are available under the terms of the Creative Commons Zero “No rights reserved” data waiver (CC0 1.0 Public domain dedication).
