# Supplementary material for: Quality parameters for RNA preparations from biopsies of ulcerated human skin
Source: Wellcome Open Res. 2023 Feb 28;7:249. Originally published 2022 Oct 5. [Version 2] doi: 10.12688/wellcomeopenres.18052.2 (PMC9984735; doi:10.12688/wellcomeopenres.18052.2)
Supplement: Peer review history

# Peer review: Reviewer response for version 2

**DOI:** 10.21956/wellcomeopenres.21193.r55319

**Published:** 2023-03-03

**Authors:** Honghua Hu

**Affiliations:** 1 Macquarie Medical School, Macquarie University, Sydney, New South Wales, Australia

The revised manuscript can be accepted.

Is the rationale for developing the new method (or application) clearly explained?

Partly

Is the description of the method technically sound?

Yes

Are the conclusions about the method and its performance adequately supported by the findings presented in the article?

Yes

If any results are presented, are all the source data underlying the results available to ensure full reproducibility?

Yes

Are sufficient details provided to allow replication of the method development and its use by others?

Yes

Reviewer Expertise:

Bacterial infections

I confirm that I have read this submission and believe that I have an appropriate level of expertise to confirm that it is of an acceptable scientific standard.

# Peer review: Reviewer response for version 1

**DOI:** 10.21956/wellcomeopenres.20019.r54050

**Published:** 2023-02-03

**Authors:** Honghua Hu

**Affiliations:** 1 Macquarie Medical School, Macquarie University, Sydney, New South Wales, Australia

The authors validated a successful method to obtain good quality RNA from ulcerated skin samples for use in translational research studies. Their results showed a biopsy size of 3 mm in diameter and preservation in Allprotect® for up to 200 days at -20°C, are best to obtain high quality RNA preparations from ulcerated skin lesion biopsy samples. The manuscript is reasonably well written and presented.

 It is a pity that the authors haven't evaluated the quality of the tissue stored in OCT media. So, the RNA quality for medium storage can't be compared. This needs to be added into the limitation of the study.

 Another limitation is that the samples size of 2mm-Allprotect® biopsies is small, only 10 samples compared with 59 samples of 3mm-Allprotect® biopsies. The discussion also needs to address why the small biopsy size affects RNA quality.

Is the rationale for developing the new method (or application) clearly explained?

Partly

Is the description of the method technically sound?

Yes

Are the conclusions about the method and its performance adequately supported by the findings presented in the article?

Yes

If any results are presented, are all the source data underlying the results available to ensure full reproducibility?

Yes

Are sufficient details provided to allow replication of the method development and its use by others?

Yes

Reviewer Expertise:

Bacterial infections

I confirm that I have read this submission and believe that I have an appropriate level of expertise to confirm that it is of an acceptable scientific standard, however I have significant reservations, as outlined above.

# Peer review: Reviewer response for version 1

**DOI:** 10.21956/wellcomeopenres.20019.r52910

**Published:** 2022-10-25

**Authors:** Ana Claudia Maretti Garcia

**Affiliations:** 1 Department of Medicine, Keck School of Medicine, University of Southern California, Los Angeles, CA, USA

In the current manuscript, the authors aimed to optimize the quality of RNA isolated from damaged skin using biopsies of varied sizes preserved in different ways. 

 The authors successfully showed that both size and preservation methods ultimately interfere with the quality and amount of RNA obtained from cutaneous lesions. They showed that 3mm biopsies preserved in Allprotect offered the best results. The authors also validated their standardized method using an extra 30 samples, proposing an efficient preservation method for skin biopsies that will be reserved for gene expression assays. 

 The manuscript is overall very organized and well-written.

Is the rationale for developing the new method (or application) clearly explained?

Yes

Is the description of the method technically sound?

Yes

Are the conclusions about the method and its performance adequately supported by the findings presented in the article?

Yes

If any results are presented, are all the source data underlying the results available to ensure full reproducibility?

Yes

Are sufficient details provided to allow replication of the method development and its use by others?

Yes

Reviewer Expertise:

Transcriptomics, molecular biology and immunology.

I confirm that I have read this submission and believe that I have an appropriate level of expertise to confirm that it is of an acceptable scientific standard.
